# COVID-19, Chloroquine Repurposing, and Cardiac Safety Concern: Chirality Might Help

**DOI:** 10.3390/molecules25081834

**Published:** 2020-04-16

**Authors:** Giovanni Lentini, Maria Maddalena Cavalluzzi, Solomon Habtemariam

**Affiliations:** 1 Department of Pharmacy–Pharmaceutical Sciences, University of Bari Aldo Moro, via E. Orabona, 4, I-70126 Bari, Italy; mariamaddalena.cavalluzzi@uniba.it; 2 Pharmacognosy Research Laboratories & Herbal Analysis Services UK, University of Greenwich, Chatham-Maritime, Kent ME4 4TB, UK; s.habtemariam@herbalanalysis.co.uk

**Keywords:** SARS-CoV-2, COVID-19, 2019-nCoV, SARS, chiral switch, MERS, Ebola, repositioning, hydroxychloroquine

## Abstract

The desperate need to find drugs for COVID-19 has indicated repurposing strategies as our quickest way to obtain efficacious medicines. One of the options under investigation is the old antimalarial drug, chloroquine, and its analog, hydroxychloroquine. Developed as synthetic succedanea of cinchona alkaloids, these chiral antimalarials are currently in use as the racemate. Besides the ethical concern related to accelerated large-scale clinical trials of drugs with unproven efficacy, the known potential detrimental cardiac effects of these drugs should also be considered. In principle, the safety profile might be ameliorated by using chloroquine/hydroxychloroquine single enantiomers in place of the racemate.

The current 2019-nCoV (SARS-CoV-2) causing COVID-19 has demonstrated that our preparedness for emerging respiratory viral infections is far too inadequate. In the absence of any known efficacious therapeutic agent or vaccine, our effective measures against the disease are somehow similar to our ancestors a century ago: constant hand washing and social distancing. The desperate need to find drugs for COVID-19 has brought the attention of numerous scholars to the repurposing of known drugs as tentative COVID-19 therapeutics.

It is logical that repurposing known drugs for COVID-19 may be first considered from pharmacological agents with proven efficacy against other highly pathogenic viral infections. However, useful medicines could also be identified from non-etiotropic drugs. In the hunt for new antiviral agents, repurposing old drugs or repositioning experimental ones are not novel approaches, and they were proposed early to treat other viral infections such as the Ebola outbreak [1]. Repurposing folk medicines was also suggested [2], while emphasis was given to cardiovascular drugs [3,4].

Accordingly, antiviral agents, cardiovascular drugs [5], and traditional Chinese remedies [6] are being considered for repurposing against the SARS-CoV-2 infection.

One of the options under investigation is the old antimalarial drug, chloroquine—a synthetic succedaneum of cinchona alkaloids [7] (Figure 1)—which gave promising results in a Chinese clinical trial, where it was superior to the positive control treatment in more than 100 patients [8]. Recently, Wang et al. have revealed that remdesivir and chloroquine are highly effective in the control of the 2019-nCoV infection in vitro and suggested that they should be assessed in human patients [9]. Further clinical evidence is needed to support these successful preliminary reports, and the hazards related to the extension of chloroquine and its analog, hydroxychloroquine (Figure 1), to a wider set of patients should be carefully evaluated [10].

Besides the ethical concern related to accelerated large-scale clinical trials of drugs with unproven efficacy [11,12], the known potential detrimental cardiac effects of chloroquine should also be taken into account [13]. Moreover, while being included in the essential medicine lists of several countries as an antimalarial [14], chloroquine may hamper cardiac function at clinically relevant doses and its safety margin is very narrow. The proarrhythmic activity of chloroquine is mainly due to its capacity to inhibit the cardiac inward rectifier potassium current and further, it can induce lethal ventricular arrhythmias. The off-target activity partially stems from the blockades of the human ether-à-go-go related gene (hERG) [15] and Kir2.1 potassium [16] channels that may occur at low μM concentrations. Additionally, while therapeutic doses of chloroquine typically result in plasma concentrations of 2–5 μM [17], peak levels up to 80 μM could be achieved in the case of a clinical overdose [18].

However, it should be noted that chloroquine is a chiral compound currently used as a racemate. It is generally accepted that stereochemistry may affect both the pharmacodynamics and ADMET of drugs [19]. Stereochemical aspects could thus be exploited to improve clinical safety and efficacy (risk-to-benefit ratio). The use of one of the separated enantiomerically pure chloroquine stereoisomers in lieu of its corresponding racemate—the so-called chiral switch approach [20]—might found its rationale in the following considerations.

Chloroquine is a moderate inhibitor of hERG with electrophysiology studies indicating IC_50_ values ranging from 2.5 to 19.7 μM, depending on the experimental setting used [21]. Site-directed mutagenesis studies revealed that chloroquine selectively blocks the above-mentioned potassium channels through structurally specific interactions. Regarding the hERG channel blockade, alanine-scanning mutagenesis and molecular docking studies indicated that chloroquine mainly interacts through specific cation-π and π-stacking interactions with Tyr-652 and Phe-656 of the proteic subunits lining the pore through a “foot in the door” type mechanism [22]. A third possible point of interaction would be offered by Ser-649 [23]. Thus, a three-point interaction binding might be envisaged and it may be anticipated that chloroquine off-target activity on cardiac functioning could display stereoselectivity. This means that one of the enantiomers could be less active as a hERG blocker and, consequently, safer to humans.

As stated above, chloroquine may prolong the QT interval of the electrocardiogram (ECG) and cause the potentially lethal long QT (LQT) syndrome (LQTS), also interfering with the normal activity of Kir2.1 potassium channels with the binding site being located in the cytoplasmic pore of the channel [16]. Selective mutation studies demonstrated that at least three residues would be involved (Glu-224 > Met-259 > Glu-299). Thus, once again, 3D requirements might cause stereoselectivity of blocks and one of the chloroquine enantiomers might display weaker interactions with respect to its optically active counterpart, thus resulting in a lesser detrimental effect on cardiac function.

ADMET aspects might also contribute to the stereoselective clinically relevant outcomes. The binding of chloroquine with human plasma protein is enantioselective with the (*S*)-enantiomer displaying higher affinity than its enantiomer [24]; further, toxicity (LD_50_) is lower for the (*S*)-enantiomer in the mouse model [25].

On the contrary, the desirable activity on COVID-19 would mostly stem from structurally unspecific aspects such as basicity and lipophilicity. The formation of SARS-CoV particles occurs in the Golgi apparatus [26]. Chloroquine is a basic tertiary amine that may enter the cell and increase the pH of acidic vesicles. Due to its relatively high lipophilicity, the non-protonated fraction of chloroquine in the biophase may easily dissolve in the phospholipidic bilayer of cell membranes and enter the cell, where it becomes protonated and accumulates in low-pH organelles, including endosomes, Golgi vesicles, and lysosomes [27]. Obviously, these processes are chirality-independent and even if both enantiomers share the same antiviral activity (regardless of their corresponding absolute configuration), the toxicologically safe enantiomer should be preferred.

Both enantiomers of chloroquine may be easily obtained from the corresponding racemate by optical resolution [28] or through stereoselective synthesis [29]. High enantiomeric purity (ep) values are mandatory for evaluating the stereoselectivity of drugs [30] and convenient methods for assessing ep values of chloroquine enantiomers are now available [31].

On the other hand, the studies on chloroquine enantiomers as antivirals might boost endeavours aimed at developing new chloroquine chiral analogs starting from this lead compound. Medicinal chemists have developed easy rules of thumb to both accelerate optimization [32] and reduce cardiac toxicity [33] by facile chemical modifications without escaping from the “drug-like” space.

Given that useful drugs for treating new diseases might stem from relatively old drugs and that chloroquine has already shown some promise in pilot COVID-19 trial studies, its full potential should be explored by testing enantiomerically pure analogs. Beyond establishing the relative potency of the enantiomers against SARS-CoV-2, their differential cardiotoxicity could lead to the identification of a more potent and safer lead.

## Figures and Tables

**Figure 1:**
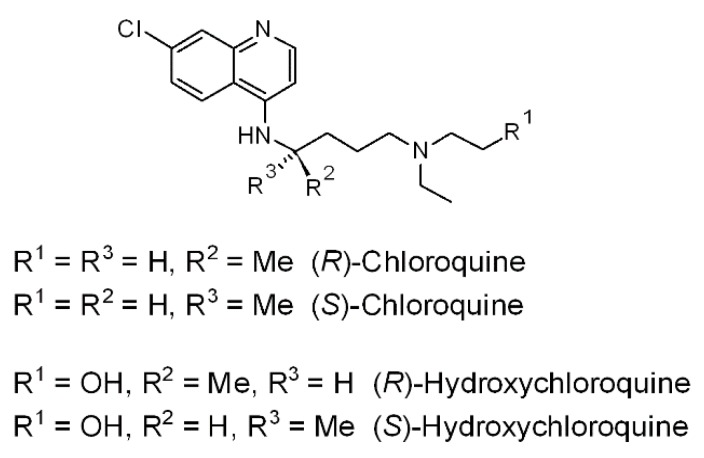
Structures of the enantiomers of chloroquine (R^1^ = H) and hydroxychloroquine (R^1^ = OH).
